# One-pot synthesis of class II lanthipeptide bovicin HJ50 via an engineered lanthipeptide synthetase

**DOI:** 10.1038/srep38630

**Published:** 2016-12-07

**Authors:** Jian Wang, Xiaoxuan Ge, Li Zhang, Kunling Teng, Jin Zhong

**Affiliations:** 1 State Key Laboratory of Microbial Resources, Institute of Microbiology, Chinese Academy of Sciences, Beijing, 100101, P. R. China; 2 University of Chinese Academy of Sciences, Beijing, 100039, P. R. China

## Abstract

Lanthipeptides are a large class of bacteria-produced, ribosomally-synthesized and post-translationally modified peptides. They are recognized as peptide antibiotics because most of them exhibit potent antimicrobial activities against Gram-positive bacteria especially those that are phylogenetically related to producers. Maturation of class II lanthipeptide like bovicin HJ50 undergoes precursor modification by LanM and a subsequent leader peptide cleavage by LanT. Herein, via co-expression of precursor gene *bovA*, modification gene *bovM* and transporter gene *bovT* in *Escherichia coli* C43 (DE3), bioactive bovicin HJ50 was successfully produced and secreted. To further achieve *in vitro* one-pot synthesis of bovicin HJ50, an engineered bovicin HJ50 synthetase BovT150M was obtained by fusing the peptidase domain of BovT (BovT150) to the N-terminus of BovM. BovT150M exhibited dual functions of precursor modification and leader peptide cleavage to release mature bovicin HJ50. Under the guidance of BovA leader peptide, BovT150M exhibited substrate tolerance to modify non-native substrates including suicin and lacticin 481. This work exemplifies the feasibility of enzyme chimera of peptidase domain (LanT150) and modification enzyme (LanM) as a one-pot lanthipeptide synthetase.

Drug-resistant bacteria have posed increasing threats to human health and have raised global concern for lack of effective resorts^1^,^2^,^3^. Lanthipeptides are polycyclic peptides featured by the presence of unusual lanthionine/methyl lanthionine and belong to a growing family of natural products known as ribosomally synthesized and post-translationally modified peptides (RiPPs)^4^. Lanthipeptides that exert antimicrobial activities are referred to lantibiotics, which are regarded as ideal alternatives to antibiotics because of their extraordinary efficacy, remarkable stability and low possibility to raise bacterial resistance^5^. Most lantibiotics act either by inhibition of cell wall biosynthesis via binding and sequestration of essential peptidoglycan precursor lipid II and/or disruption of the membrane integrity via pore formation^4^,^6^. Nisin, the prototype lanthipeptide, has been long used as food preservative worldwide for over 50 years without occurrence of bacteria resistance and has been in clinical trials to treat diseases like bovine mastitis^7^. Recent genome-based mining over increasing microbial genomes envisioned the unanticipated wide distribution of lanthipeptide gene clusters, which greatly outnumbered known lanthipeptides and are thus a fascinating arsenal for peptide antibiotics^8^.

Lanthipeptides are produced exclusively by Gram-positive bacteria and their biosynthesis-related genes are assembled in gene clusters encoding precursor peptides (LanA), modification enzymes (LanBC/LanM/LanKC/LanL), transporters (LanT), processing proteases (LanP), immunity proteins (LanFEG/LanH/LanI) and regulation machineries. Posttranslational modification of precursor peptide and subsequent leader peptide cleavage are the most pivotal steps for lanthipeptide maturation. Initially, precursor LanA is ribosomally produced as a linear peptide composed of an N-terminal leader peptide and a C-terminal core peptide. Under the guidance of leader peptide, the modification enzyme executes a sequential dehydration and cyclization process, during which certain Ser or Thr residues in the core peptide are firstly dehydrated and then cyclized with Cys residues to form intramolecular thioether bridges^9^. Removal of the N-terminal leader peptide by protease is required to release bioactive product referred as lanthipeptide. Lanthipeptides are mainly divided into four classes based on the diversity of modification enzymes^10^. Class II lanthipeptides, one of the most extensively investigated family members of lanthipeptides, are modified by a bifunctional LanM which contains an N-terminal dehydratase domain and a C-terminal LanC-like cyclase domain^8^. After precursor modification, the leader peptides of class II lanthipeptides are in most cases processed by LanT, which is an ABC transporter with an N-terminal cysteine peptidase domain and a C-terminal ATP-binding domain^11^. LanT is responsible for cleavage of the leader peptide at a highly conserved double glycine motif (GlyGly/GlyAla/GlySer), concomitant with exporting the mature lanthipeptides outside the cell^12^. While in a few cases, a second trimming of an additional hexa-peptide is required by a serine protease LanP like lichenicidin and cerecidin^13^,^14^.

Traditional methods to discover novel lanthipeptides are mainly via bioactivity-based screening of producer strains or heterologous expression of lanthipeptide gene clusters in related model hosts, which are somewhat time-consuming and cost-extensive^15^,^16^. Thus, efficient and potent synthetic strategy would no doubt facilitate expedient generation and bioengineering of lanthipeptides. Reconstitution of LctM, the synthetase of a typical class II lanthipeptide lacticin 481, unlocked the gate to *in vitro* bioengineering of lanthipeptides^17^. Until recently, this *in vitro* modification strategy combined with leader peptide cleavage by commercial protease was fully exploited in lanthipeptide biosynthesis^18^,^19^. Bovicin HJ50 (PDB ID: 2M8V), produced by *Streptococcus bovis* HJ50, is reminiscent of lacticin 481 but differed by an unusual disulfide bridge^20^,^21^,^22^. Bovicin HJ50 contains a conserved putative lipid II binding motif that indicates its mode of action by inhibiting cell wall biosynthesis as well as pore formation on membrane^21^. The gene cluster for bovicin HJ50 biosynthesis includes 9 genes namely *bovAMTFEGKRI*^23^,^24^. Bovicin HJ50 has been produced by way of a two-step semi-*in vitro* biosynthesis system (SIVB) consisting of (1) *in vivo* modification of precursor peptide by co-expression of precursor gene *bovA* and modification gene *bovM* in *E. coli* and (2) *in vitro* digestion of leader peptide by N-terminal peptidase domain of BovT (BovT150) (Fig. 1a)^20^. Thus, the reconstituted BovM and BovT150 could function as the minimal machinery for bovicin HJ50 biosynthesis.

In the present study, we established a one-pot synthesis system for bovicin HJ50. First, we achieved bovicin HJ50 production in *E. coli* via co-expression of *bovA* and *bovM* with either *bovT*_*150*_ or intact *bovT*. Prompted by that, an engineered lanthipeptide synthetase BovT150M (BovT150-BovM fusion enzyme) was successfully reconstituted that exerted dual functions of precursor modification and leader peptide cleavage. Bovicin HJ50 as well as bovicin HJ50-like lanthipeptide suicin, were successfully produced via BovT150M. This work provided a new approach to release bioactive lanthipeptides via a modification and processing enzyme chimera, which could be potentially applied for expedient biosynthesis and bioengineering of novel lanthipeptides.

## Results

### One-pot synthesis of bovicin HJ50 *in vivo*

BovA could be successfully modified by BovM when they are co-expressed in *E. coli*^20^. To exploit *E. coli* as microbial biofactory for bovicin HJ50 production, *bovA* and *bovM* were co-expressed with N-terminal peptidase *bovT*_*150*_or intact *bovT*. Expression of single *bovA* or co-expression of *bovA* and *bovM* in *E. coli* BL21 (DE3) could not produce any antimicrobial activities when assayed against sensitive indicator strain *Micrococcus flavus* NCIB8166 (Fig. 2a). However, when *bovA, bovM* and *bovT*_*150*_ were co-expressed, the cell lysates that were induced for more than 10 h exhibited antimicrobial activity, whereas the supernatants exhibited no activity (Fig. 2a). MALDI-TOF MS analysis of the cell lysates detected the presence of bovicin HJ50 with m/z as 3428.6 Da (Fig. 2b). Co-expression of *bovA, bovM* and *bovT* in *E. coli* BL21 (DE3) did not produce any bioactive agents in neither supernatants nor cell lysates (Fig. 2a). This indicated that the peptidase domain BovT150 was capable to cleave the leader peptide of BovM modified precursor *in vivo*, whereas the full length BovT might not be functionally expressed in *E. coli* BL21 (DE3).

To reconstitute the full length BovT *in vivo*, we adopted a host strain *E. coli* C43 (DE3), a BL21 (DE3) derived strain suitable for membrane protein expression^25^. Interestingly, co-expression of *bovA, bovM* and *bovT* in *E. coli* C43 (DE3) produced bioactive bovicin HJ50 in supernatants but not in cell lysates, which was further confirmed by MS analysis (Fig. 2c). This indicated that mature bovicin HJ50 was produced and exported outside from *E. coli* cells. Thus, bioactive lanthipeptide bovicin HJ50 was successfully produced in *E. coli* via manipulating the minimal biosynthetic machinery.

### One-pot synthesis of bovicin HJ50 via engineered BovT150M

Prompted by the concomitant functionality of BovM and BovT150 in *E. coli*, we created a recombinant bovicin HJ50 synthetase BovT150M (BovT150-BovM) by fusing the BovT150 to the N-terminus of BovM. To reconstitute the *in vivo* function of the engineered BovT150M, *bovA* was co-expressed with *bovT*_*150*_*M* in *E. coli* BL21 (DE3) via transformation with pET28a-*bovA* and pACYC-Duet-*bovT*_*150*_*M*. When induced by IPTG, bovicin HJ50 was produced *in vivo* but not exported outside the cell membrane as only cell lysates showed antimicrobial activity (Fig. 2a). This indicated that BovT150M might exert precursor modification and leader peptide digestion functions simultaneously. Quantification of bovicin HJ50 in the cell lysates using agar diffusion bioassay showed that 0.20 μg/ml to 0.91 μg/ml bovicin HJ50 were produced (Supplementary Information, Figure S1). Cell growth curve indicated that *in vivo* production of bovicin HJ50 led to slight growth retardation (Supplementary Information, Figure S1).

To reconstitute BovT150M *in vitro*, BovT150M was expressed and purified by immobilized ion metal affinity chromatography (IMAC) (Supplementary Information, Figure S2a). Precursor hexahistidine-tagged BovA (His_6_-BovA) was expressed and purified from inclusion bodies by IMAC and C18 reversed phase high performance liquid chromatography (RP-HPLC) as described previously^26^. When incubating His_6_-BovA (20 μM) and BovT150M (2 μM) in assay buffer containing 10 mM Mg^2+^, 1 mM DL-dithiothreitol (DTT) and 2.5 mM adenosine triphosphate (ATP) for 1 h, bioactive bovicin HJ50 was produced (Fig. 3a). MS analysis of the product exhibited an [M+H]^+^ of 3430.6 Da, which was in good accordance with authentic bovicin HJ50 with an unfolded disulfide bridge (Fig. 3a).

Reductive agents like DTT are supposed to be required for the catalytic function of BovT150M in that BovT150 is a cysteine protease and BovM also needs reductive conditions to modify substrate BovA^24^. When DTT was absent, incubation of His_6_-BovA and BovT150M for more than 4 h could not produce any bioactive agents (Fig. 3b). MS analysis showed the mass peak of 8275.7 Da, which was about 4 Da decrease compared with calculated mass of 8279.3 Da of His_6_-BovA (Fig. 3b). This indicated that 2 disulfide bridges were spontaneously formed in His_6_-BovA and BovT150M could hardly modify or digest the disulfide cross-linked His_6_-BovA as indicated in previous research^17^,^24^. Although reductive conditions are required for the fusion enzyme to correctly modify and release bovicin HJ50, the unfolded disulfide bridge could be spontaneously re-formed when DTT was removed or in other oxidative conditions^24^. ATP was also demonstrated to be indispensable for the catalytic function of BovT150M as absence of ATP eliminated the dehydration activity of BovT150M (Fig. 3c). ATP might serve as phosphate donors during the phosphorylation of substrate BovA by BovM domain^17^.

### Mutational analysis of BovT150M to elucidate its functionality

The leader peptide has been indicated to be nonessential for modification function of LanM because LctM can still produce partially processed LctA in the absence of leader peptide^27^. However, without the leader peptide, the modification activity of LanM was greatly impaired^28^,^29^. Recently, an engineered leader-LctM fusion enzyme LctCE was generated that was constitutively active to modify LctA core peptide to produce authentic lacticin 481 but with limited efficacy^29^. Herein, BovT150M could efficiently produce fully modified bovicin HJ50 when incubated with His_6_-BovA, suggesting that the leader peptide directed precursor modification by BovM domain might commence before leader peptide cleavage by BovT150 domain.

**Figure 1:**
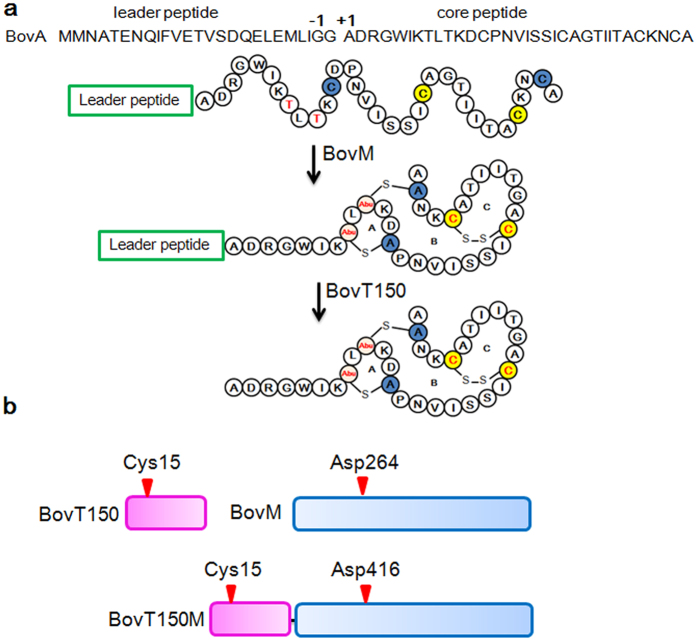
Biosynthesis of bovicin HJ50 and crucial residues in BovT150, BovM and BovT150M. (**a**) Maturation of bovicin HJ50 requires modification by BovM and leader peptide cleavage at the double glycine motif by BovT150. (**b**) BovT150 is a cysteine peptidase containing a crucial Cys15 and BovM is the modification enzyme of bovicin HJ50 containing a crucial Asp264. The engineered bovicin HJ50 synthetase BovT150M contains an N-terminal BovT150 and a C-terminal BovM, in which Cys15 corresponds to Cys15 in BvoT and Asp416 corresponds to Asp264 in BovM.

**Figure 2:**
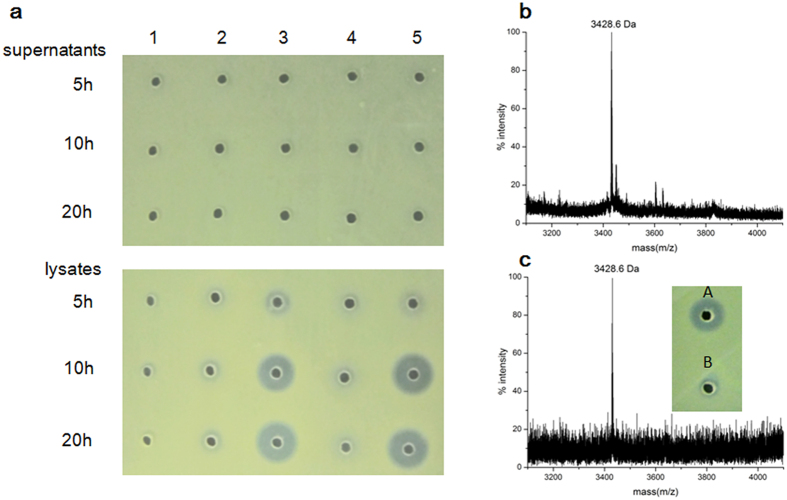
Production of bovicin HJ50 in *E. coli*. (**a**) Antimicrobial assay of supernatants and cell lysates of *E. coli* BL21 (DE3) after induced by IPTG for 5, 10, 20 h. *E. coli* cells are transformed with (1) pET28a-*bovA*; (2) pET28a-*bovAM*; (3) pET28a-*bovAM* + pACYC-Duet-*bovT*_*150*_; (4) pET28a-*bovAM* + pACYC-Duet-*bovT*; (5) pET28a-*bovA* + pACYC-Duet-*bovT*_*150*_*M. M. flavus* NCIB8166 was used as the indicator strain for antimicrobial assay. (**b**) MS analysis of cell lysates of *E. coli* BL21 (DE3) co-transformed with pET28a-*bovA* and pACYC-Duet-*bovT*_*150*_*M*. (**c**) MS analysis and antimicrobial assay of supernatants of *E. coli* C43 (DE3) co-transformed with pET28a-*bovAM* and pACYC-Duet-*bovT* after induced by IPTG. A, culture supernatants; B, cell lysates.

**Figure 3:**
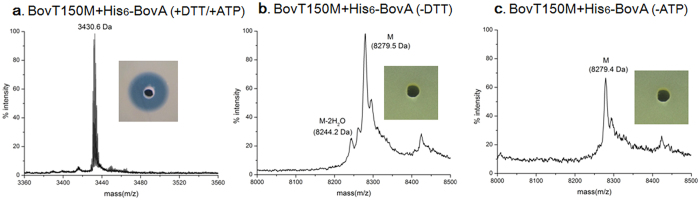
Enzymatic activity assay of the engineered bovicin HJ50 synthetase BovT150M. MS analysis and antimicrobial assay of reaction products after incubation of His_6_-BovA and BovT150M in the presence of DTT and ATP (**a**), in the absence of DTT (**b**), and in the absence of ATP (**c**).

**Figure 4:**
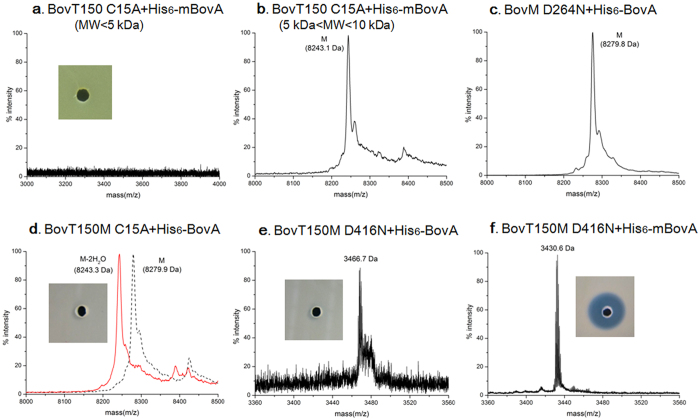
Mutagenesis of BovT150M. MS analysis and antimicrobial assay of reaction products after incubation of His_6_-mBovA and BovT150 C15A (**a**,**b**), His_6_-BovA and BovM D264N (**c**), His_6_-BovA and BovT150M C15A (**d**), His_6_-BovA and BovT150M D416N (**e**), His_6_-mBovA and BovT150M D416N (**f**). In panel d, dotted line indicated the MS spectrum of unmodified His_6_-BovA and red line indicated the MS spectrum of His_6_-BovA treated by BovT150M C15A.

**Figure 5:**
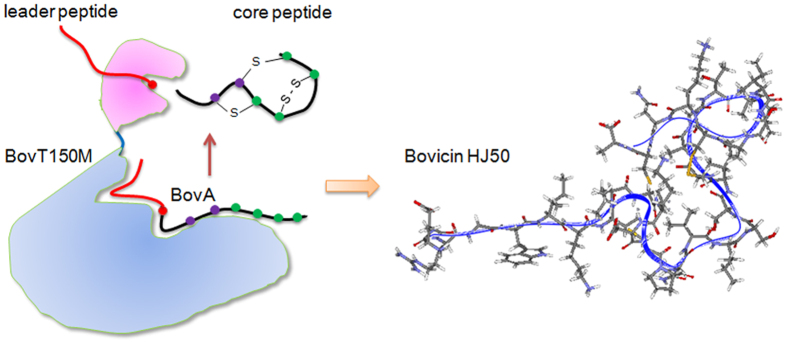
Proposed model for the catalytic mechanism of engineered BovT150M. Pink and light blue represent BovT150 domain and BovM domain, respectively. Red and black lines represent leader peptide and core peptide of BovA, respectively. BovA core peptide is firstly modified by BovM domain to introduce thioether rings under the guidance of BovA leader peptide. Then mBovA is digested by BovT150 domain at the double glycine motif to release bioactive bovicin HJ50.

To demonstrate this hypothesis, we eliminated either peptidase or modification function via mutation of active sites in BovT150M. Cys15 is the enzymatic center of peptidase BovT150 while Asp264 of BovM are crucial and conserved in LanM proteins (Fig. 1b)^30^,^31^,^32^. As expected, BovT150 C15A was unable to cleave leader peptide of modified BovA (His_6_-mBovA) while BovM D264N was unable to modify His_6_-BovA even in 4 h (Fig. 4a,b and c). However, incubation of His_6_-BovA with BovT150M C15A produced fully modified His_6_-mBovA (m/z 8243.3 Da) but not active bovicin HJ50, indicating that BovT150M C15A maintained modification function but abolished peptidase activity (Fig. 4d). BovT150M D416N, corresponding to D264N mutation in BovM domain, could not modify His_6_-BovA while only trace amounts of unmodified core peptide was produced after 4 h (Fig. 4e). BovT150M D416N actually retained efficient peptidase activity towards modified precursor His_6_-mBovA as bioactive bovicin HJ50 (m/z 3430.6 Da) was produced in 1 h (Fig. 4f). The mutagenesis analyses indicated that BovT150 domain and BovM domain could function independently whereas loss-of-function of BovM domain will significantly impair the proteolytic activity of BovT150 domain towards unmodified precursor His_6_-BovA. Thus we proposed a successive working mode for BovT150M that leader peptide guided precursor modification via BovM domain precedes leader peptide proteolysis via BovT150 domain (Fig. 5).

### One-pot synthesis of other lanthipeptides via BovT150M

**Figure 6:**
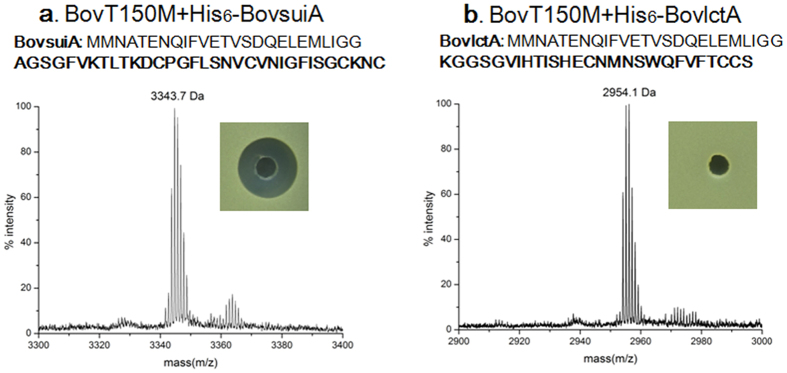
Production of suicin and lacticin 481 using BovT150M. MS analysis and antimicrobial assay after incubation of His_6_-BovsuiA with BovT150M (**a**) and incubation of His_6_-BovlctA with BovT150M (**b**).

To test the generality of BovT150M on substrates other than bovicin HJ50, two short peptides BovsuiA and BovlctA were designed and expressed as chimeras with BovA leader peptide at the N-terminus (Supplementary Information, Figure S2b). Suicin was a bovicin HJ50-like lanthipeptide restored from a remnant *lan* locus of *S. suis* serotype 2 and lacticin 481 was a typical class II lanthipeptide with no disulfide bridge^26^. The chimeric peptide His_6_-BovsuiA consisting of BovA leader peptide and suicin core peptide was purified and incubated with BovT150M. Antimicrobial assay indicated that the reaction product was inhibitory against *M. flavus* NCIB8166 and MS analysis showed the mass peak of 3343.7 Da (Fig. 6a), which was in good accordance with authentic suicin with unfolded disulfide bridge. Chimeric peptide His_6_-BovlctA (lacticin 481) consisting of BovA leader peptide and lacticin 481 core peptide was expressed, purified and subjected to BovT150M. MS analysis of the reaction product showed a mass peak of 2954.1 Da, which was 18.2 Da decrease compared with calculated mass of lacticin 481 core peptide (2972.3 Da) (Fig. 6b). This indicated that lacticin 481 was one-fold dehydrated by BovT150M. Antimicrobial assay indicated that the modified product was inactive (Fig. 6b). Thus, BovT150M was capable of fully modifying and generating bioactive bovicin HJ50-like lanthipeptides while partially modifying other class II lanthipeptides like lacticin 481. However, dehydration of non-native lanthipeptides by BovT150M implied the substrate tolerance of BovM domain and the capability of BovT150 domain to release the core peptides that were appended to BovA leader peptide.

## Discussion

Lanthipeptides are a fast-growing class of gene-encoded and ribosomally-synthesized peptides with multi-functions, most of which are conferred with potent antimicrobial activities against Gram-positive bacteria. Because of their high efficacy and stability, lanthipeptides are regarded as promising candidates for novel antimicrobial applications in many areas like food preservatives and antibiotics^6^. With accumulating elucidation of lanthipeptide biosynthetic pathways and modification machineries, genome-based mining enabled revealing of lanthipeptide repertoire in a wider variety of species than anticipated^8^,^33^,^34^. However, isolation of lanthipeptides from natural resources is a tremendous work, let alone certain lanthipeptide clusters are even cryptic or conditionally expressed^13^,^26^,^35^.

To facilitate bioengineering of lanthipeptides, we first achieved lanthipeptide production in a model biofactory like *E. coli.* Bovicin HJ50, a typical class II lanthipeptide, was produced in *E. coli* C43 (DE3) via introduction of minimal biosynthetic genes *bovA, bovM* and *bovT*. Recently, production of the two-component lanthipeptide lichenicidin (Bliα and Bliβ) has also been achieved in *E. coli* via co-expression of *licA, licM, licT* and/or *licP*^36^. Thus, it is feasible to manipulate minimal genetic prerequisites in *E. coli* to obtain bioactive lanthipeptides. However, the growth retardation of bovicin HJ50 producing cells indicated that bovicin HJ50 might interfere with the cell wall synthesis by sequestration of intracellular lipid II.

The successful production of bovicin HJ50 via co-expression of *bovA, bovM* and *bovT150* in *E. coli* cells was encouraging, as BovA could be processed by BovM and BovT150 simultaneously to release bioactive bovicin HJ50. Prompted by that, we reconstituted an engineered lanthipeptide synthetase BovT150M by fusing the peptidase domain BovT150 to the N-terminus of the modification enzyme BovM. A similar approach fused the leader peptide of lacticin 481 or Halβ to their corresponding modification enzyme LctM or HalM2, generating constitutively active lanthipeptide synthetases LctCE or HalCE2 but with limited efficiency^29^,^37^. The leader peptide was important for precursor modifications due to its role in LanM recognition and binding^28^,^37^. With intact leader peptide, the modification activity of BovM domain towards precursor was fully maintained. Additionally, fusing protease domain BovT150 to BovM could facilitate the stability of BovT150, which was unstable in *in vitro* conditions as also observed in LctT150 30. Thus, BovT150M facilitated both precursor modification and leader peptide cleavage. Our results further demonstrated that the separate domains of BovT150M exerted functions successively; first, the leader peptide guided precursor modification via BovM domain and then the leader peptide was cleaved by BovT150 domain. One interesting finding was that a novel LanT and LanM fusion protein (SBI_06987) was recently identified in *Streptomyces bingchenggensis* BCW-1 genome, suggesting the possible co-functionality of LanT and LanM proteins in native microbes^38^.

The fusion lanthipeptide synthetase could be applied for efficient and rapid one-pot synthesis of lanthipeptides. We focused on using BovT150M to generate class II lanthipeptides that are similar to bovicin HJ50. Under the guidance of BovA leader peptide, authentic suicin is obtained while lacticin 481 is one-fold dehydrated but not bioactive. Suicin is a bovicin HJ50-like lanthipeptide with two thioether bridges (ring A and B) and a disulfide bridge, while lacticin 481 has three thioether bridges instead. All of them share a conserved ring A structure, which is the proposed lipid II binding motif with two dehydratable Thr/Ser. This also demonstrated that the modification machinery of bovicin HJ50 was different from that of lacticin 481, though bovicin HJ50 is structurally resembled with lacticin 481 with an N-terminal linear and C-terminal globular structure. Furthermore, the potential application of this lanthipeptide synthetase approach may be extended to introducing non-native thioether rings or nonproteinogenic amino acids into short artificial peptides^39^,^40^.

In conclusion, we achieved bovicin HJ50 production in *E. coli* via co-expression of minimal biosynthetic genes. Specifically, an engineered lanthipeptide synthetase BovT150M was reconstituted both *in vivo* and *in vitro* to produce mature bovicin HJ50. This one-pot synthesis system provides new options for production and *in vitro* bioengineering of novel lanthipeptides. Moreover, BovT150M implies potential application in introducing dehydro amino acids or thioether bridges into non-native substrate peptide drugs, which might enhance thermostability or maintain structural conformation.

## Materials and Methods

### Materials

*Escherichia coli* DH5α was used for plasmid construction and *E. coli* BL21 (DE3) and C43 (DE3) for protein expression. Plasmid pET28a and pACYC-Duet-1 were used as expression vectors. Kanamycin of 50 μg/ml and chloramphenicol of 10 μg/ml were used when needed. *E. coli* strains were incubated in Luria-Bertani (LB) medium at 37 °C and *Micrococcus flavus* NCIB8166 was inoculated in S1 medium at 30 °C^13^.

### Cloning, Mutagenesis and Protein Expression

Molecular biology methods were performed according to standard protocols^41^. Plasmid pET28a-*bovA*, pET28a-*bovAM* and pET28a-*bovT*_*150*_ were constructed previously^20^,^24^. *bovT*_*150*_ and *bovT* were amplified from genomic DNA of *S. bovis* HJ50 with primers containing *Nde*I and *Kpn*I and were then respectively constructed into pACYC-Duet-1 to obtain pACYC-Duet-*bovT*_*150*_ and pACYC-Duet-*bovT. bovT*_*150*_ with non-stop codon was constructed into pACYC-Duet-1 between *Nde*I and *Kpn*I and *bovM* was ligated between *Kpn*I and *Xho*I subsequently. This plasmid was named pACYC-Duet-*bovT*_*150*_*M*. pET28a-*bovT*_*150*_*M* was obtained by constructing *bovT*_*150*_*M* into pET28a. Chimeric genes *bovsuiA* and *bovlctA* were synthesized by Sango Biotech (Shanghai, China) and constructed into pET28a, respectively. Site-directed ligase-independent mutagenesis (SLIM) was performed to introduce mutations by a PCR method as described by *Chiu*^42^. Protein expression and purification were conducted as described previously^26^. Purified proteins were identified by 16% acrylamide SDS-PAGE and protein concentrations were determined by BCA assay kit (Thermo Scientific, USA) according to instructions.

### Enzyme Activity Assay

The reaction buffer (50 mM Tris-HCl, 150 mM NaCl, 10 mM MgCl_2_, pH 7.4) was used for activity assay of BovT150 and 1 mM DTT and 2.5 mM ATP were needed for BovM and BovT150M. His_6_-BovA, BovM and BovT150M were respectively used with a final concentration of 20 μM, 2 μM and 2 μM. The reactions were proceeded at 25 °C for 1 h to 4 h and quenched by 0.5% trifluoroacetic Acid (TFA). The reaction products were analyzed by mass spectrometry (MS) analysis.

### Bioactivity Detection

Antimicrobial activity was determined by well-diffusion method against indicator strain *M. flavus* NCIB8166. 25 μl samples of culture supernatants, cell lysates or *in vitro* biosynthesized products were applied to wells with diameter of 5 mm on agar plates containing *M. flavus* NCIB8166 and the agar plates were incubated in 30 °C for 24 h. Concentration of bovicin HJ50 from cell lysates were determined by measuring the diameter of inhibition zone with agar diffusion bioassay as described by *Pongtharangkul*^43^. *E. coli* BL21 (DE3) containing pET28a-*bovA* and pACYC-Duet-*bovT*_*150*_*M* were induced by 0.5 mM IPTG. Cell growth curve were recorded by measuring OD600 and induced *E. coli* cells of 5 ml were pelleted and resuspended in 1 ml PBS solution, and further lysed via sonication. The lysates were centrifuged and 25 μl aliquot was loaded to the agar plate containing sensitive indicator strain *M. flavus* NCIB8166. Purified bovicin HJ50 via SIVB was diluted into a gradient concentration of 20, 10, 5, 2.5, 1.25 and 0.625 μg/ml, which were used as for construction of the standard curve.

### MS Analysis

MALDI-TOF (matrix-assisted laser desorption/ionization-time of flight) MS analysis was performed on 4700 Proteomics Analyzer mass spectrometer (Applied Biosystems, USA). Samples were prepared by acidification with adding 0.1% TFA and subsequent processing via C18 ZipTip column. CHCA (α-cyano-4-hydroxycinnamic acid) matrix was prepared by dissolving 5 mg in 1 ml of 50:50 acetonitrile/water containing 0.1% TFA. Mass spectra for 1–5 kDa were obtained in positive reflectron mode and 5–10 kDa in linear mode.

## Additional Information

**How to cite this article**: Wang, J. *et al*. One-pot synthesis of class II lanthipeptide bovicin HJ50 via an engineered lanthipeptide synthetase. *Sci. Rep.* **6**, 38630; doi: 10.1038/srep38630 (2016).

**Publisher's note:** Springer Nature remains neutral with regard to jurisdictional claims in published maps and institutional affiliations.

## Supplementary Material

Supplementary Information
