# Supplementary material for: One-pot synthesis of class II lanthipeptide bovicin HJ50 via an engineered lanthipeptide synthetase
Source: Sci Rep. 2016 Dec 7;6:38630. doi: 10.1038/srep38630 (PMC5141572; doi:10.1038/srep38630)
Supplement: Supplementary Information [file srep38630-s1.pdf]

Supplementary Information

One-pot synthesis of class II lanthipeptide bovicin HJ50 via an engineered lanthipeptide synthetase

Jian Wang[1, 2], Xiaoxuan Ge[1, 2], Li Zhang[1, 2], Kunling Teng[1], Jin Zhong[1,*].

[1] State Key Laboratory of Microbial Resources, Institute of Microbiology, Chinese Academy of Sciences, Beijing 100101, PR China.

[2] University of Chinese Academy of Sciences, Beijing 100039, PR China.

* Corresponding author. E-mail: zhongj@im.ac.cn

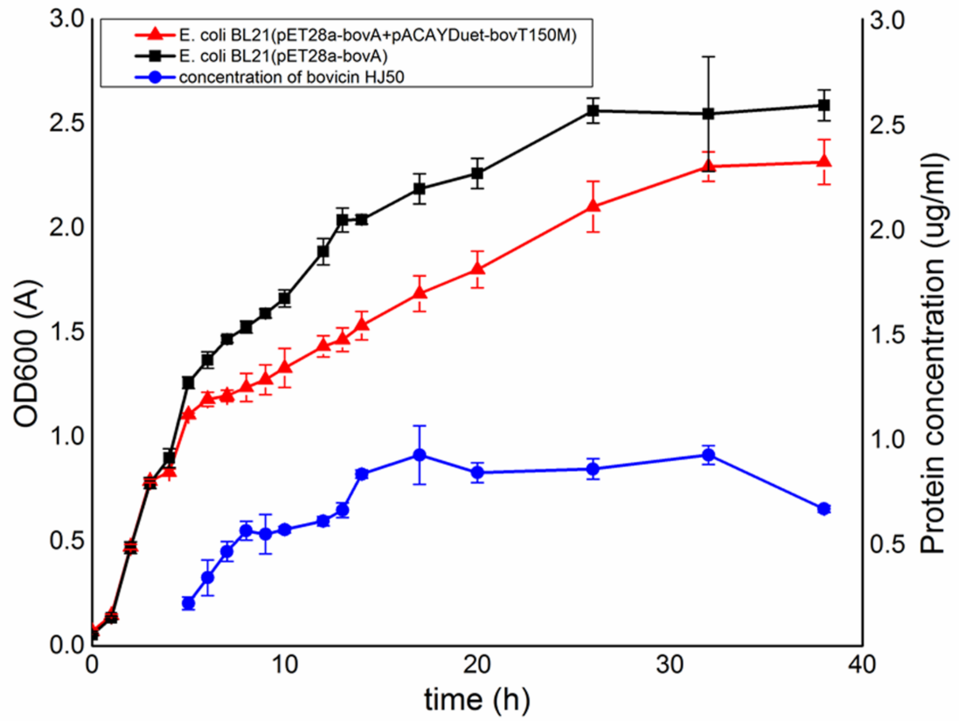

Figure S1. Growth curve of *E. coli* cells and bovicin HJ50 production in the cell lysates. Black line, growth curve of *E. coli* BL21 (DE3) containing plasmid pET28a-*bovA*; red line, growth curve of *E. coli* BL21 (DE3) containing plasmids pET28a-*bovA* and pACYC-Duet-*bovT*$_{150}$*M*; blue line, concentration of bovicin HJ50 in the cell lysates of *E. coli* BL21 (DE3) containing plasmids pET28a-*bovA* and pACYC-Duet-*bovT*$_{150}$*M*. IPTG was added at 3 h and triplicates were recorded.

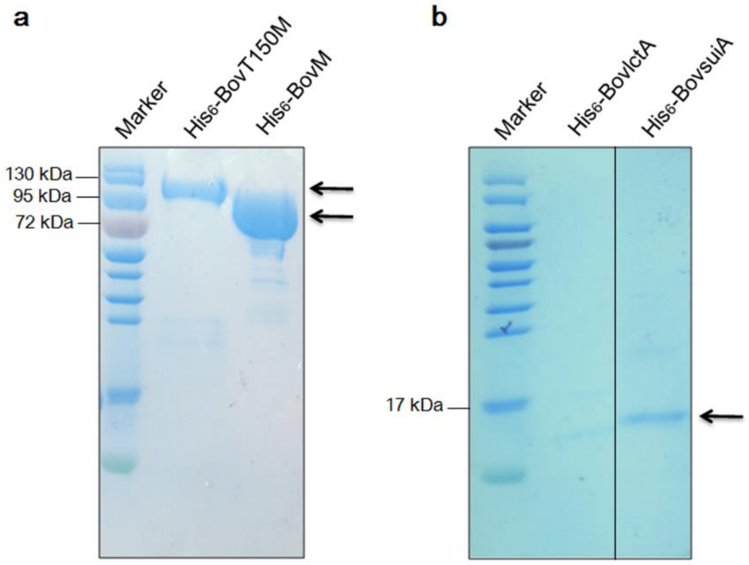

Figure S2. Identification of purified proteins by SDS-PAGE. (a) Identification of His$_6$-tagged BovT150M (118.8 kDa) and BovM (101.2 kDa). (b) Purification and identification of His$_6$-tagged BovlctA and BovsuiA.

Amino acid sequence of recombinant protein BovT150M. The His_6 tag was colord in gray, the BovT150 domain was colored in yellow and the BovM domain was colored in green. The crucial Cys15 of the enzymatic center of BovT150 domain and the conserved Asp264 of BovM domain are shown in red.

MGSSHHHHHHSSGLVPRGSHMASMTGGQQMGRGSMRKIKEEIQFQQNDCGP
CCLSMILNYYDYQITVPELSSQLSKKNGWSMLDLKTVAAKYYLQSNVFKITD
LKGFDKLQCPLVLFWESSHFVVLESISENIVIVDPSIGHVKLNRNEFNQKFSGF
AMSFVKNEHFQKRKLSKLRYVKEFNKAGTMEKLDLTINNESESFHKIIDNIVS
DFYLVILDAVQTNAELSNVHKYLYKNIRQVLLPILVQDINEWRLESKHQKNDT
NQEYIDYCYLFISKNRFAYLKNKYELLNLRIDTIISETKLNLKNFLKNIDKSVSS
LKKVFPQCDFEIKKLKFIDFIGDNHGLYQSIMFEVSGKVFFYKCHGSEITNFIVT
LQKEIPSLNFLKLPMTYIDQEFIIQEKVTHSSVLIKDDIEEYYHNMGKLLGILYL
LNGNDMHNENIIARGKNPIVIDVETLINPIECDSDSKMDDSIFSVGMLPMKYN
RNFEGIFDTSSLGQSNIVSEKVFKEYKPFTSEIQLVLIDHTFSDLDRYLPRYKEIH
FDVIDYLGVVEDGFIESYHLIMNHKKEIAKVISREASNINCRIVLRNTRIYSEIL
KFLSTPSLLKNITKAEDTLKRLLVKPRYIIHNWERYRQQEIKQLLNGEIPYFSFG
KYGNTSPNDLNLSSVETSLQCKLNRFSDDDLKFQLILLRTSLNIDDDNFEIIGKE
NFRGKLKDLIKSSIYGTDIFTLQKDWCGNYIYGESNLGLYEGKLGIILSDSYFS
DIFDVSSIRREILFSRDISFIDGRASLDVFDIINNDSILSPVSVEISNKYLDLIEGIS
GVILVRYKFYCQKLPENISELEQLMRQFMRLYKNSSELKNGFAHGISGIKIIFYI
AYQILGEEKYLDEFKKLEILDFPEKYTNGSWCNGLLGWITSEYILYTISGDKTF
KENILNHLPRLMTYLIDLSDYCLCHGSFGVLDFLLTLSQKELLPKEFHDEYVK
LEKYHLEMITQCRILKKDVSLFTGISGILYYLNRKESGKNSVLTFGF.
